# Bioactive Components and Health Potential of Endophytic Micro-Fungal Diversity in Medicinal Plants

**DOI:** 10.3390/antibiotics11111533

**Published:** 2022-11-02

**Authors:** Sundaram Muthukrishnan, Paranivasakam Prakathi, Thangavel Sivakumar, Muthu Thiruvengadam, Bindhu Jayaprakash, Venkidasamy Baskar, Maksim Rebezov, Marina Derkho, Gokhan Zengin, Mohammad Ali Shariati

**Affiliations:** 1 Department of Biotechnology, Ayya Nadar Janaki Ammal College, Sivakasi 626124, Tamil Nadu, India; 2 Department of Microbiology, Ayya Nadar Janaki Ammal College, Sivakasi 626124, Tamil Nadu, India; 3 Department of Crop Science, College of Sanghuh Life Sciences, Konkuk University, Seoul 05029, Korea; 4 Departmentof Biotechnology, Sri Shakthi Institute of Engineering and Technology, Coimbatore 641062, Tamil Nadu, India; 5 Department of Oral and Maxillofacial Surgery, Saveetha Dental College and Hospitals, Saveetha Institute of Medical and Technical Sciences, Saveetha University, Chennai 600077, Tamil Nadu, India; 6 Department of Scientific Research, V. M. Gorbatov Federal Research Center for Food Systems, 26 Talalikhin Str., Moscow 109316, Russia; 7 Department of Scientific Research, K. G. Razumovsky Moscow State University of Technologies and Management (The First Cossack University), 73 Zemlyanoy Val, Moscow 109004, Russia; 8 Department of Scientific Research, Russian State Agrarian University—Moscow Timiryazev Agricultural Academy, 49 Timiryazevskaya Str., Moscow 127550, Russia; 9 Department of Natural Sciences, South-Urals State Agrarian University, 13 Gagarin Str., Troitsk 457100, Russia; 10 Department of Biology, Science Faculty, Selcuk University, Konya 42130, Turkey

**Keywords:** entophytic fungi, bioactive compounds, antibacterial, antioxidant, molecular docking, GC-MS, FTIR

## Abstract

The endophytic fungi that reside inside medicinal plants have the potential to produce various pharmaco-potential bioactive compounds. The endophytic fungi *Graminicolous helminthosporium*, *Bipolaris australiensis* and *Cladosporium cladosporioides* were isolated from different medicinal plants. The GC-MS analysis of intra- and extracellular products of endophytic fungi revealed the presence of various bioactive metabolites, such as Anthracene, Brallobarbital, Benzo [h] quinolone, Ethylacridine, 2-Ethylacridine, Cyclotrisiloxane, 5 methyl 2 phenylindolizine, and 1,4-Cyclohexadien-1-one, etc. The phytochemical composition analysis of endophytic fungus extracts also revealed the presence of flavonoids, phenols, saponins, carbohydrates, glycosides, and proteins. The intra- and extracellular endophytic extracts exhibited strong antibacterial and antioxidant activity, which was screened with the agar-well diffusion method and DPPH, H_2_O_2_, and nitric oxide scavenging activity, respectively. The bioactive compounds identified in the endophytic extracts from GC-MS profiling served as ligands for molecular-docking analysis to investigate the anticancer potential against non-small cell lung carcinoma receptor EGFR. Molecular docking results showed that compounds, such as Brallobarbital, and 5 methyl 2 phenylindolizine had the lowest E- min values, which suggests that these compounds could be used in anticancer drug development. Thus, the isolated endophytic fungal species can be used to produce various bioactive compounds that could be used in novel drug development from natural sources and reduce the environmental burden of synthetic chemical drugs.

## 1. Introduction

Endophytes are the microorganisms present in the innermost part of the plant ecosystem and play a vital role in their mechanisms. Most endophytes are isolated from the plant parts, such as the leaf, stem, root, flower, meristem, and seeds. Endophytes are plant mutualists living asymptomatically within plant tissues [1]. Most classes of plants with lignified tissues are commonly used for the isolation of host-dependent endophytes. In initial research, the isolated endophytes from the temperate zone grasses commonly belong to the family Clavicipitaceae. There is evidence that endophytes induce the host’s resistance capacity against pathogens, climatic changes, natural changes, artificial changes, and disease. The isolated plant endophytes also have the potential to produce compounds of an anti-pathogenic nature in humans when an endophyte has a long-term association with the plant, which has the ability to be involved in the plant’s metabolic pathways and biosynthetic activity [2].

Endophytes isolated from medicinal plants can produce a high range of functionally active metabolites. Endophytic fungi produce bioactive secondary metabolites, including phytohormones [3]. Endophytic fungi are one of the prime resources for obtaining bioactive metabolites due to their complex interactions with their host plants or other microbes within the host plants [4,5]. Many plant species also use endophytes to regulate their life cycles, in areas such as defense systems, regulatory mechanisms, nutrient supply, and growth. Endophytic fungi produce different secondary metabolites based on environmental factors and the host system. Endophytes are able to produce secondary metabolites; these may be an antibiotic, therapeutic agents, agrochemicals, and enzymes [4]. Endophytes are rich in bioactive compounds that actively participate in industrial, biomedicinal, and agricultural processes. Endophytes are mostly unexplored, but they are the prime sources of antioxidant, insecticidal, antitumor, antidiabetic, immunosuppressive, and biocontrol compounds [4,5].

The extraction of secondary metabolites as well as bioactive compounds from endophytic fungi has been conducted with the help of the liquid-liquid extraction method. Liquid extraction is a method of extracting samples into immiscible aqueous and organic solutions by partition [6]. Inorganic solvents were used for the methodological extraction of the sample after proper shaking and separation techniques. Larger volumes of solvents are required in this liquid-liquid extraction process because this method consumes more time. FTIR and GC-MS are commonly employed methods to determine bioactive compounds within fungal samples [6].

Many bacterial species are noted to be most dangerous to humans and have emerged as antibiotic-resistant species. Some of these are *Pseudomonas aeruginosa*, *Escherichia coli, Klebsiella pneumoniae*, and *Streptococcus pyogenes*, etc., The agar well diffusion and agar disc methods are routinely utilized to examine the antibacterial efficacy of the extracted compounds. The agar well diffusion method is well-known to be used to test the antimicrobial activity of any kind of extract from a sample [7].

Cell damage is widely caused by the effects of oxidative stress, which is controlled by the antioxidant activities of several natural compounds. Oxidative stress could increase factors, such as aging, with pathologies that cause infection or inflammation and neurological diseases that affect the autoimmune system. Phytochemicals from non-nutritive have the ability to decrease the incidence of disease [8]. In recent research, both men and women were highly affected by lung cancer, and fewer treatments were available in the current situation. Non-small cell lung cancer (NSCLC) causes severe lung carcinoma, but the signaling pathway of the NSCLC and its control method was determined. The expression caused frequent lymph node metastasis, poor chemosensitivity and a low survival rate of EGFRs in NSCLC. EGFRs from the erbB family (erbB1, erbB2 (HER2), erbB3, and erbB4) were related to receptor tyrosine kinases and their pathways. After the binding of EGFR with the ligand, the phosphorylation process begins in the intracellular domain, leading to the downstream signal transduction pathway [8].

The present work focused on the isolation of endophytic micro-fungi and the screening of their bioactive compounds by FTIR and GC-MS analysis. Furthermore, biochemical tests for protein, carbohydrates, flavonoids, tannins and alkaloids were employed to detect the presence of various phytoconstituents. Antibacterial and antioxidant activities were carried out to determine the bioactive potentials of the intra- and extracellular fungal extracts. In addition, molecular docking studies were employed to determine the anticancer properties of the identified compounds in the extracts.

## 2. Materials and Methods

### 2.1. Sample Collection

**Figure 1:**
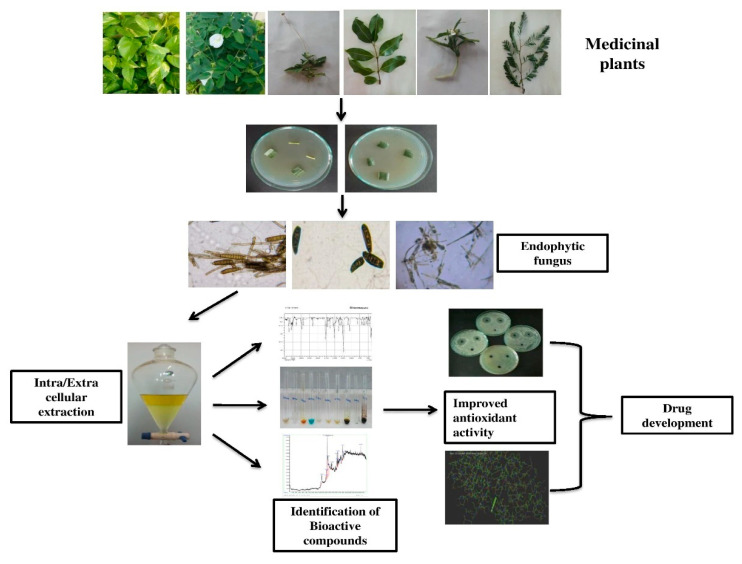
A schematic representation of the workflow of the current study.

Plants with medicinal properties were collected, originating from Sivakasi (9.4533° N, 77.8024° E). Ten plant samples, namely, *Leucas aspera, Tamarindus indica, Jatropha gossypifolia, Senna auriculata, Manikara zapota, Clitoriaternatea, Portulaca grandiflora, epipremnumaureum, Tridax procumbens* and *Acalypha indica* with medicinal values were collected and placed in separate sterile zip lock bags followed by processing within 24 h of collection. To avoid contamination, the fresh plant leaf and stem were taken for the isolation process. The above medicinal plants have already been used for their antifungal, prostaglandin inhibitory, antioxidant, antimicrobial, antinociceptive and cytotoxic activities, and anticancer properties, within cases of wound curing, abdominal pain, diarrhea, dysentery, parasitic infection, fever, malaria and respiratory problems [9]. The workflow of the present work is represented in Figure 1.

### 2.2. Surface Sterilization

The dust and debris attached to the samples were removed by washing them in running water. Then the samples were sliced into tiny pieces of 1 cm, followed by washing two to three times with sterile distilled water before they were taken into a laminar airflow chamber. First, the samples were rinsed with sodium hypochlorite solution (5%) and then cleaned with distilled water twice. The samples were treated with 70% ethanol and cleaned with sterile distilled water. After that, the samples were subjected to 1% mercuric chloride and washed with sterile distilled water. Once again, the samples were washed with 79% ethanol for two minutes and then washed with sterile distilled water twice. The surface-sterilized samples were placed on sterile tissue paper to remove excess moisture or water in the samples [10].

### 2.3. Sample Inoculation

The inoculation media was prepared with Potato Dextrose Agar (PDA) medium or Sabouraud Dextrose Agar (SDA) medium or Malt Extract Agar (MEA) medium. These media were prepared and sterilized in an autoclave for 20 min at 121 °C. While transferring the medium to a sterile Petri plate, antibiotics, such as tetracycline and amoxicillin, were included at a palm-bearable temperature to reduce Gram-positive and Gram-negative bacterial contamination. After solidification, surface-sterilized samples were inoculated with the help of clean forceps. The inoculated samples were incubated at 28 °C for 15 days [11].

### 2.4. Morphological Identification of Endophytic Fungi

The staining solutions, such as Lactophenol and Lactophenol cotton blue stain, were procured from Hi Media Laboratories Private Limited, India. The slides prepared were sealed with DPX mountant. Identification of the fungal species was conducted as prescribed in, “The Genera of Hyphomycetes from the soil” [12], in the Compendium of soil fungi [13].

### 2.5. Lactophenol Cotton Blue Mounting

With the use of a clean inoculation loop, a small amount of culture was taken, and semi-permanent slides were developed with lactophenol cotton blue. The slides were mildly heated in a spirit lamp to remove the air bubbles from inside the cover glass. The surplus dye was discharged using tissue paper, and the cover glass was sealed with white nail polish.

### 2.6. Diversity Analysis

A fungal colony from the inoculated plant samples was used for the diversity analysis, which was stained and examined under a microscope. The white fungal strains were stained with lactophenol cotton blue dye, and dark color sporing, such as black or grey strains, were stained with paraffin oil. The fungal strain was taken with a sterile needle and placed on the microscopic slides loaded with a few drops of stain. Then the fungal strain in the slides was subjected to teasing to spread the conidia and spores of the fungi and then examined under a microscope [11].

### 2.7. Production of Fermentation Medium

The fungal strain was transferred to the production medium for the production of secondary metabolites. The fermentation medium was prepared with a combination of 50% potato extract and a 5% carbon source, such as dextrose, along with 20% of mineral sources, such as yeast extract, dipotassium hydrogen orthophosphate, magnesium sulphate with heptahydrate, sodium chloride, and ferrous sulphate. The production medium above this concentration was prepared in 200 mL of a 500 mL Erlenmeyer flask and autoclaved to maintain a sterile environment. After the production medium attained room temperature, antibiotics, such as tetracycline and amoxicillin, were added to it at a concentration of 200 mg/L. The fungal strain with spores was transferred to the prepared fermentation medium and incubated at 28 °C for 15 days [9].

### 2.8. Extraction of Metabolites

The extraction of secondary metabolites was the major process to screen out the bioactive compounds produced by the endophytic fungi present in the production medium. The liquid-liquid extraction method was utilized to extract the metabolites (intra/extracellular) from the production medium. For the extracellular metabolites, the liquid broth of the production medium was collected after 20 days of incubation. Initially, the production medium was filtered using a glass funnel and filter paper. The filtrate could not contain any mycelium or fungal culture. This filtrate was mixed with an equal amount of ethyl acetate and allowed to incubate for four hours at room temperature. Then the filtrate and ethyl acetate mixture were partitioned with a separating funnel. The organic layer was collected and used for the screening process of bioactive compounds [9]. For the intracellular metabolites, the mycelium from the production medium was harvested and disrupted with mortar and pestle, along with the solvent methanol. The ground mycelium extract was filtered with a grass cloth. The extract was lyophilized and then analyzed with the instruments, such as FTIR and GC-MS [14].

### 2.9. FTIR Analysis

The endophytic fungal extracts were analyzed with FTIR to predict the functional group present in the metabolites. The absorption and transmittance range used in the FTIR was 400 to 4000 cm^−1^. The sample was mixed with powdered KBr and exhibited to a hydraulic pellet press to prepare the pellet for analysis. Then the peak range of the fungal samples was used to predict the functional groups [15].

### 2.10. GC-MS Analysis

Identification of bioactive compounds in the fungal extract of both extracellular and intercellular was conducted using GC-MS. The liquid sample without any contaminant or debris was used for the test sample of GC-MS. To achieve this, the test samples were centrifuged at 3000 rpm for 5 min. The clear supernatant was taken as the test sample. The sample powder was dissolved with methanol and centrifuged for the intracellular extract to obtain a clear supernatant [15].

### 2.11. Antibacterial Assay

The agar-well diffusion method was used to test the antibacterial activity of the test samples. The nutrient medium was prepared with an additional agar source with a concentration of 2%. The medium was sterilized with an autoclave. The Petri plates with nutrient agar were inoculated with bacterial cultures, such as *Escherichia coli, Streptococcus pneumoniae, Shigella sps,* and *Streptomyces avermitilis.* Then, with the sterile well puncher, a well with a diameter of 1 cm was created on the cultured agar plates. The test samples with different concentrations of 10 µL and 20 µL were added to the well in the Petri plate. The solvent used to dissolve or extract the fungal metabolites was used as the control for this agar-well diffusion method. The control solvents were ethyl acetate and methanol. The zone of inhibition was recorded after incubation to forecast the ability of the sample against bacterial colony growth [16].

### 2.12. Phytochemical Analysis

Both the extracellular and intracellular extracts were used for the phytochemical analysis. Ten phyto-compounds were analyzed in this work, such as flavonoids, alkaloids, saponins, tannins, carbohydrates, terpenoids, steroids, phenols, glycosides, and proteins [17].

### 2.13. Flavonoids Test

#### 2.13.1. Alkaline

A total of 2 mL of the fungal extract was added to 2 to 3 drops of 2% diluted NaOH solution. The yellow color change of the test indicated the presence of flavonoids [17].

#### 2.13.2. Lead Acetate

A total of 2 mL of the extract was taken, and a few drops of lead acetate was added to it. The yellow color precipitate formation in the test indicated the presence of flavonoids in the extract [17].

#### 2.13.3. Ferric Chloride

A total of 2 mL of the fungal extract was taken, and a few drops of ferric chloride were added to it. The green color change of the test indicated the presence of flavonoids [17].

#### 2.13.4. Sulphuric Acid

A total of 2 mL of the fungal extract was taken, adding a few drops of concentrated H_2_SO_4_. The formation of an orange color in the test indicated the presence of flavonoids [17].

#### 2.13.5. Saponins

A total of 1 mL of the fungal extract was taken and mixed with 5 mL of distilled water. The test tube was mixed efficiently for 15 to 20 s, followed by maintaining at room temperature for 10 min. The generation of a stable form indicated the presence of saponins in the fungal extract [17].

### 2.14. Tannins Test

#### 2.14.1. Ferric Chloride

A total of 2 mL of the fungal extract was taken along with 1 mL of 5% FeCl_2_. The color change indicated the presence of tannin in the sample. The dark green-black color formation indicated the presence of condensed tannins, and the dark blue-black color formation indicated the presence of hydrolyzed tannins in the sample [17].

#### 2.14.2. Gelatin

A total of 2 mL of the fungal extract was taken along with 1% gelatin solution, and 1 mL of 10% NaCl was added. The white color precipitate formation in the test indicated the presence of tannin in the sample [17].

#### 2.14.3. Lead Acetate

A total of 2 mL of the fungal extract was taken, adding a few drops of lead acetate. The formation of a white color precipitate in the test indicated the presence of tannin in the sample [17].

### 2.15. Proteins Test

#### 2.15.1. Millon

A total of 2 mL of the fungal extract was taken along with 2 mL of the Millon reagent. The formation of a white color precipitate in the test indicated the presence of proteins in the sample [17].

#### 2.15.2. Biuret

A total of 2 mL of the fungal extract was taken. A drop of 2% CuSO_4_ and 1 mL of ethanol and a sodium hydroxide pellet was added to the extract, followed by incubation for 2 to 5 min without shaking. The formation of a pink color indicated the presence of protein in the sample [17].

#### 2.15.3. Ninhydrin

A total of 2 mL of the fungal extract and 2 mL of the ninhydrin solution were taken in the test tube and placed in a water bath for 5 min at 50 °C. The formation of violet-blue or violet color in the test indicated the presence of protein in the solution [17].

#### 2.15.4. Aminoacids

A total of 2 mL of the fungal extract was taken along with a few drops of lead acetate and diluted NaOH. The formation of a black color precipitate indicated the presence of protein in the sample [17].

### 2.16. Carbohydrate Test

#### 2.16.1. Benedicts

A total of 2 mL of the fungal extract was taken along with 2 mL of Benedict reagent, then was placed in a water bath for 2 min at 50 °C. The formation of the orange and red colors indicated the presence of carbohydrates in the sample [17].

#### 2.16.2. Iodine

A total of 2 mL of the fungal extract was taken along with 2 mL of the iodine solution. The formation of dark blue or purple color indicated the presence of carbohydrates in the sample [17].

#### 2.16.3. Fehlings

Initially, the Fehling A and Fehling B solutions were diluted well and boiled for 1 min. After boiling, the clear blue-colored Fehling solution was obtained. A total of 2 mL of the fungal extract was taken along with 1 mL of the prepared Fehling solution. The test sample was placed in a water bath for 5 min at 50 °C. The formation of a dark brick-red color indicated the presence of carbohydrates in the sample [17].

#### 2.16.4. Terpenoids

A total of 2 mL of the fungal extract was taken along with 2 mL of chloroform. Then 2 mL of con. H_2_SO_4_ was added to the test tubes. The test sample was placed in a water bath for 2 min at 50 °C. The formation of a grey color suggested the presence of terpenoids in the sample [17].

#### 2.16.5. Steroids

A total of 2 mL of the fungal extract was taken along with a few drops of chloroform and 1 mL of the con. H_2_SO_4_. The production of the brown ring specified the presence of steroids, and the emergence of the bluish-brown ring suggested the presence of phyto-steroids [17].

#### 2.16.6. Glycosides

A total of 2 mL of the fungal extract was taken along with a few drops of FeCl_2_ as well as 1 mL of glacial acetic acid, and 1 mL of the con. H_2_SO_4_. The production of the brown ring indicated the presence of cardiac glycosides in the sample [17].

#### 2.16.7. Alkaloids

##### Mayer Test

A total of 2 mL of the fungal extract was taken with a few drops of Mayer reagent and 1 mL of the con. H_2_SO_4_ was added. The formation of yellow color in the test indicated the presence of alkaloids in the sample [17].

#### 2.16.8. Phenols

##### Ferric Chloride Test

A total of 2 mL of the fungal extract was taken along with 3–4 drops of 10% ferric chloride solution and 1 mL of potassium ferro cyanide. The formation of a bluish-black color in the test indicated the presence of phenols in the sample [17].

##### Litmus Paper Test

The red color changes in the litmus paper confirmed the presence of phenols in the sample when the litmus paper was dipped in the sample to be tested [17].

### 2.17. Antioxidant Analysis

The fungal species contained a property to inhibit or reduce the cell detriment imposed by free radicals or other unstable molecules. The quantitative presence of antioxidant properties in the endophytic fungal extract was estimated by DPPH, H_2_O_2_, and nitric oxide scavenging analysis [18].

#### 2.17.1. DPPH Radial Scavenging Activity

For DPPH scavenging activity, the test samples were taken at different concentrations from 10 µL to 50 µL. Then the test samples were made up to 100 µL with DPPH solution. After incubation, the violet color changed to yellow based on the concentration, indicating the physical presence of the antioxidant property. The test samples were examined under UV spectrophotometry with absorption of 517 nm. The standard was prepared with ascorbic acid. The standard was also taken at different concentrations, from 10 µL to 50 µL and made up with DPPH up to 100 µL, followed by incubation for up to 30 min at 37 °C. After incubation, the standard was examined under UV spectrophotometry with an absorption of 517 nm. Pure methanol was used as a blank, whereas the 3 mL DPPH solution was used as a control for DPPH scavenging activity [18]. The percentage of DPPH was calculated with the following equation.

$$\mathrm{DPPH}\;\left(\mathrm{scavenging}\;\mathrm{effect}\;\%\right)=\frac{{\mathrm{Abs}}_{\mathrm{control}}-{\mathrm{Abs}}_{\mathrm{sample}}}{{\mathrm{Abs}}_{\mathrm{control}}}\times 100$$

where the Abs_control_ and Abs_sample_ were the absorption of control and absorption of the sample at 517 nm.

#### 2.17.2. H_2_O_2_ Radical Scavenging Activity

The test samples were taken at different concentrations of 10 µL to 50 µLand were made up to 100 µL with H_2_O_2_ solution (4 mM H_2_O_2_ was dissolved in phosphate buffer). The test sample was examined under a UV spectrophotometer with the absorption at 230 nm. Pure methanol was used as the control. The standard (ascorbic acid) was also prepared as a test sample and incubated for up to 10 min at 37 °C. After incubation, the standard was examined under UV spectrophotometry with absorption at 230 nm [18]. Pure methanol was used as the blank, whereas the 3 mL pure hydrogen peroxide solution was used as a control. The percentage of the H_2_O_2_ scavenging activity was calculated with the following equation.

$$H_{2}O_{2}\;\left(\mathrm{scavenging}\;\mathrm{effect}\;\%\right)=\frac{{\mathrm{Abs}}_{\mathrm{control}}-{\mathrm{Abs}}_{\mathrm{sample}}}{{\mathrm{Abs}}_{\mathrm{control}}}\times 100$$

whereas the Abs_control_ and Abs_sample_ were the absorption of control and absorption of the sample at 230 nm.

#### 2.17.3. Nitric Oxide Scavenging Activity

A nitric oxide scavenging assay was performed as reported previously by Palanichamy et al. [2]. The test sample and standard (ascorbic acid) was taken atdifferent concentrations of 10 µL to 50 µl and made up with methanol solution up to 100 µL. Then 1.5 mL of 10 mM sodium nitroprusside was added to all the tubes, then the reaction was incubated at 25 °C for 30 min. After incubation, the samples were equally mixed with the Griess reagent and subsequently examined under UV spectrophotometry with an absorption of 546 nm. Pure methanol was used as the blank, whereas the 3 mL of sodium nitroprusside and Griess reagent mixture was used as a control for nitric oxide scavenging activity [18]. The percentage of nitric oxide scavenging activity was calculated with the below equation.

$$\mathrm{Nitric}\;\mathrm{oxide}\;\left(\mathrm{scavenging}\;\mathrm{effect}\;\%\right)=\frac{{\mathrm{Abs}}_{\mathrm{control}}-{\mathrm{Abs}}_{\mathrm{sample}}}{{\mathrm{Abs}}_{\mathrm{control}}}\times 100$$

where the Abs_control_ and Abs_sample_ were the absorption of control and absorption of the sample at 546 nm.

### 2.18. Molecular Docking

EGFR kinase (Epidermal Growth Factor Receptor kinase) is the receptor for non-small cell lung cancer (NSCLC), which has a high risk of death. The entire description of the receptor was collected from the site of the Therapeutic Target Database. The 3D structure of EGFR for humans was retrieved from the PDB database as PDB ID—1M14 (EGFR_HUMAN). The PDB form of the receptor was obtained by Online Smile Translator and transferred to the docking tool Hex 8.0. Then the ligand for docking was chosen by the peak range of GC-MS. The molecular structure of the ligand was obtained from the PubChem database, and the PDB form of the 3D structure of the ligand was retrieved from Online Smile Translator and transferred to the docking tool Hex 8.0. The docking was done with Hex 8.0 with receptors and ligands. The Hex message with E-minimum values was observed to predict the interaction of the ligand with receptor EGFR [19].

### 2.19. FT-IR Spectrophotometer Analysis

Infrared spectra (IR) were also used to identify the bioactive compounds. Micro-fungal extracts were tested using a SHIMADZU-FT-IR instrument to analyze the functional groups in the bioactive constituents.

### 2.20. GC–MS Analysis

The microfungal bioactive compounds were analyzed by a gas chromatograph (GC-2010) interfaced using a quadrupole mass spectrometer (QP-2010) analyzer to examine its chemical constituents using Rtx-5 capillary column (30 m ×0.32 mm ×0.5 μm).

### 2.21. Statistical Analysis

All the trials were carried out in triplicate with mean standard deviation (SD). Each experiment was repeated twice with the level of significance at *p* ≤ 0.05. Means were separated using Duncan’s multiple range test.

## 3. Results and Discussion

### 3.1. Endophytic Microfungal Isolation

Endophytic fungi naturally occur in plant tissue and play an essential role in plant development by the secretion of novel bioactive compounds, phytopathogenic activity, and phytohormones. Khalil et al. [20] reported that endophytes, such as *penicillium crustosum, Penicillium chrysogenum, Penicillium commune, Penicillium corylophilum, Alternaria infectoria, Penicillium caseifulvum, Alternaria alternata, Alternaria tenuissima, Aspergillus flavus,* and *Aspergillus niger* were isolated from the plant, *Ephedra pachyclada*. Similarly, Saroj et al. [21] demonstrated the isolation of endophytic microfungi as *Bipolarisaustraliensis* from *Senna angustifolia* plants in India.

**Figure 2:**
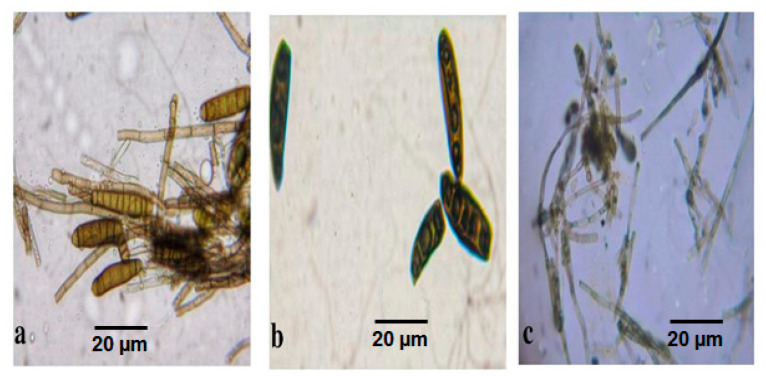
Morphological characteristics of endophytic fungi. Microscopic aspect of hyphae and spores of endophyte isolated from (**a**) *Graminicolous helminthosporium*, (**b**) *Bipolaris australiensis*, and (**c**) *Cladosporium cladosporioides*. Scale bars = 20 µm.

Jannath et al. [22] reported that the medicinal plant *Baliospermum montanum* (Willd.) Muell was distributed throughout India, and twenty-nine endophytic fungal species were isolated from the tissues of this medicinal plant. The phytochemical analysis recorded that 70% of endophytic isolates showed alkaloids and flavonoids, and 13% were positive for phenols, saponins, and terpenoids. GC-MS profile detected twenty-five bioactive compounds from ethyl acetate extracts. Among endophytic fungi, *Trichoderma reesei* isolated from the flowers exhibited nine bioactive compounds, namely 2-Cyclopentenone, 2-(4-chloroanilino)-4-piperidino, Oxime-methoxy-Phenyl, Methanamine N-hydroxy-N-methyl, Strychane, Cyclotetrasiloxane, Octamethyl, and 1-Acetyl-20a-hydroxy-16-methylene [22]. The endophytic bioactive compounds are explored to develop a novel drug with commercial value. Similarly, in the present study, three species of endophytic fungi, such as *Graminicolous helminthosporium, Bipolaris australiensis* and *Cladosporium cladosporioides* were spotted and isolated from the leaves and stems of medicinal plant samples, such as *Leucas aspera, Tamarindus indica, Jatropha gossypifolia, Senna auriculata, Manikara zapota, Clitoriaternatea, Portulaca grandiflora, Epipremnumaureum, Tridax procumbens,* and *Acalypha indica*. The endophytic fungi emerged from the plant sample after 18 days of incubation. Different staining methods using paraffin oil and lactophenol cotton blue dyes displayed diversified endophytic fungi under microscopical observation. The mature *Graminicolous helminthosporium* was a greenish-black color colony with dark color spores in the SDA medium. The microscopic examination of *G. helminthosporium* showed a brown color elongated spore with ten segments along with conidia (Figure 2a). The mature colony of *Bipolaris australiensis* was a blackish brown colony with brown color spores. The microscopic examination of *Bipolaris australiensis* showed brown color oval-shaped spores with five segments along with curved conidia (Figure 2b). The mature colony of *Cladosporium cladosporioides* was a grayish black colored small colony with brown color spores. *Cladosporium cladosporioides* grew slower and formed a small colony in MEA medium. The microscopic examination of *Cladosporium cladosporioides* showed that it was grey in color, with tiny round spores with segmented conidia (Figure 2c).

More than 15 bioactive compounds were recorded in the extracellular and intracellular products of the isolated endophytic micro-fungi, namely *Graminicolous helminthosporium, Bipolaris australiensis,* and *Cladosporium cladosporioides*. The endophytic extract comprised the bioactive compounds, such as Benzo [h] quinoline, 2-Ethylacridine, Cyclotrisiloxane, Anthracene, 5-methyl-2-phenylindolizine, Indole-2-one, Tris (tert-butyldimethylsilyloxy)arsane, 3-quinolinecarboxylic acid, Benzene, n-Hexadeconic acid, Oleic acid, Oxacyclotetradecan-2-one, Cis-Vaccenic acid, Oxacyclotetradecan-2-one, Cis-Vaccenic acid, and Tetrasiloxane.

*Aspergillus brasiliensis*, which was isolated from a root, and *Fusarium oxysporum*, which was isolated from seed, each produced nine and seven bioactive chemicals from the endophyte. Overall, it was found that the various endophytic fungi associated with *B. montanum* contributed significantly to the production of bioactive chemicals, which might be useful for the potential development of novel drugs with marketable benefits [22]. Similarly obtained endophytic bioactive compounds possess various medicinal and industrial values, such as antitumor, antioxidant, wound healing, antibacterial, antimalarial, antiviral, anticonvulsant, anti-HIV, anti-inflammatory, reduction of coronary heart disease (CHD), industrial manufacturing dyes, lubricant, drug development, fuel, anti-polymerization agent, insecticidal, wood preservative, preparation of cool-tar, fossil fuel, and cosmetics.

### 3.2. FTIR Analysis

Sundar et al. [23] reported the prediction of metabolites of *Cladosporium* endophytic fungi of both (extracellular) ethyl acetate or dichloro-methane extract and (intracellular) methanol mat based on functional groups using FTIR. Elango et al. [15] found that the ethyl acetate crude extract of *Aspergillus sojae* was analyzed with FTIR and recorded the functional groups, such as N-N stretching, C-H stretching, O-H stretching, CH_2_ bending, and C-H bending in the different peaks of absorption. In accordance with this, in our study, functional groups in the fungal extracts, such as methanol mat (intracellular) and ethyl acetate (extracellular) of *Graminicolous helminthosporium, Bipolaris australiensis,* and *Cladosporium cladosporioides* were analyzed by FTIR and recorded many functional groups in the extracellular extract of *Cladosporium cladosporioides* which included C-O stretching and C-H stretching as a major functional group. In *Bipolaris australiensis* CO-O-CO stretching, C-O stretching, N-O stretching, and C=O stretching wereidentified as the major functional groups. In *Graminicolous helminthosporium,* the predicted major functional groups were CO-O-CO stretching, C-N stretching, S=O stretching, N-O stretching, and C=O stretching. The analysis of intracellular methanol mat extract from *Graminicolous helminthosporium* revealed major functional groups, such as O-H bending, C=C stretching, and C-H stretching. While in *Bipolaris australiensis* the noticed major functional groups were C-Br stretching, C-Br stretching, C=C bending, S=O stretching, and N=O stretching were the major functional groups identified in FTIR. In *Cladosporium cladosporioides* the observed functional groups were CO-O-CO stretching, S=O stretching, O-H bending, C=C stretching, O-H stretching, and N-H stretching. Mehdi et al. [24] reported that the FT-IR spectrum confirmed the presence of the alcohol group, alkene group, amine group, carbonates, ethers, carboxylic acid, and disulfides in leaf extracts of *Tamarindus indica*. Similarly, in our study, we noticed the alkene, aldehyde, alcohol, anhydride, and amine groups. Different peaks of absorption were obtained in this study, and the functional groups were tabulated (Table 1).

### 3.3. GC-MS Analysis for Bioactive Compound Screening

Recently, Tran et al. [25] reported three bioactive compounds, such as maydisone, 7-hydroxy-2,5-dimethylchromone, and 2,5-dimethylbenzoic acid in microfungal species of *Bipolaris* by using GC-MS analysis. Similarly, the bioactive compounds in three endophytic micro-fungal species, such as *Graminicolous helminthosporium, Bipolaris australiensis,* and *Cladosporium cladosporioides* were analyzed by GC-MS analysis. Bioactive compounds, such as 1,4-bis (trimethylsilyl) benzene, Arsenous acid, 1,2-bis (trimethylsilyl) benzene, Tris (tert-butyldimethylsilyloxy) arsane, Cyclotrisiloxane, and 2-Ethylacridine were commonly present in three fungal species of the ethyl acetate and methanol extract of endophytic micro fungal species. The endophytes create a variety of anti-cancerous compounds, such as taxol which has great potential to be used as an anticancer metabolite [26]. Because of this, there has been plenty of opportunity to use these metabolites against breast, uterine, and ovarian cancers [27]. These discoveries have shed light on endophytic fungi and their metabolites, which have the potential to be effective treatments for a variety of diseases. An endophytic fungus *Phoma medicaginis* associated with the medicinal plants *Medicago sativa* and *Medicago lupulina* produced the antibiotic brefeldine A, which also initiated apoptosis in cancer cells [28]. About 1500 fungal metabolites have shown antitumor and antibiotic activity [29], and some have been approved as drugs. Endophytes synthesize bioactive metabolites that may be involved in the host-endophyte relationship [26] and may function as primary sources of novel natural products for exploitation in medicine, agriculture, and industry [30]. Kanjana et al. [31] reported the ethyl acetate extracts of *Chaetomium globosum*, *Cladosporium tenuissimum* and *Penicillium janthinellumn* were shown to contain Hexadecanoic acid, Octadecanoic acid, Octadecanoic acid, 9-Octadecenoic acid, and 9,12-Octadecadienoic acid. Similarly, in our study, Benzo [h] quinoline was observed in the ethyl acetate extract of *Bipolaris australiensis, Cladosporium cladosporioides,* and the methanol extract of *Cladosporium cladosporioides.* The metabolites, such as n-Hexadeconic acid, and Cis-Vaccenic acid were noted in the ethyl acetate extract of *Bipolaris australiensis.*

Interestingly, the following bioactive compounds, namely n-Hexadeconic acid, Oleic acid, Oxacyclotetradecan-2-one, Octadec-9-ecoin acid, Cis-Vaccenic acid, Cis-13-Octadeconic acid, Octadeconic acid, Trichloroacetic acid, Bromoacetic acid, 13-Octadecenal, Ethyl 2(2-chloroacetamido)-3,3,3-trifluro-2-(3-fluroanilino) propionate, 3-(4,7-Dimethylocta-3,7-dienyl)-1H-indole, Octasiloxane, heptasiloxane, cyclopentene, tetrasiloxane, 1,4-Cyclohexadien-1-one, and Brallobarbital were found in *Bipolaris australiensis* extracts whereas the essential bioactive compound, Benzo [h] quinolone is present in the ethyl acetate and methanol mat extracts of *Cladosporium cladosporioides.* Anthracene is a major bioactive compound present in two endophytic microfungal species, such as *B. australiensis,* and *Cladosporium cladosporioides.* The bioactive compound 5-methyl-2-phenylindolizine was observed in two endophytic microfungal species, namely *G. helminthosporium,* and *B.australiensis.*

Hateet [32] reported that an endophytic fungus (*Acremonium coenophialum*) was isolated from the medicinal plant *Myrtus communis,* and the microfungal extracts were analyzed by GC-MS to determine the chemical composition of fungal extract and their results showed the presence of nineteen compounds. In line with this, GC-MS analysis of the ethyl acetate and methanol mat extract of *Graminicolous helminthosporium, Bipolaris australiensis* and *Cladosporium cladosporioides* revealed several bioactive compounds, which was presented in Table 2 and the Supplementary Files.

### 3.4. Antibacterial Potential of the Microfungal Extract

**Figure 3:**
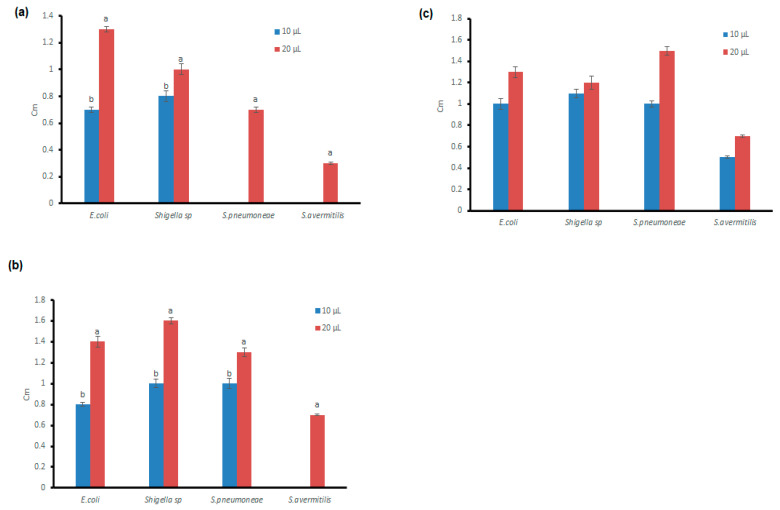
Antibacterial activity of the ethyl acetate (extracellular) extract of (**a**) *Cladosporium cladosporioides,* (**b**) *Graminicolous helminthosporium,* and (**c**) *Bipolaris australiensis* against various bacterial pathogens. Data are represented as means± SD of three replicates. If followed by different letters, the results are significantly different according to Duncan’s multiple range test at *p* ≤ 0.05.

**Figure 4:**
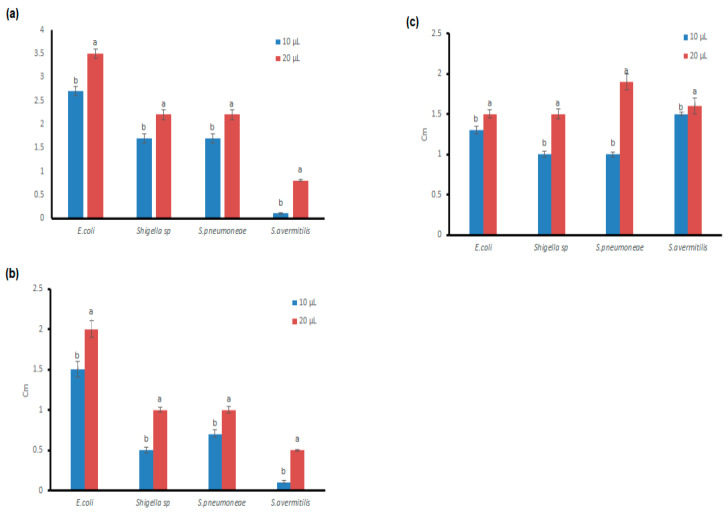
Antibacterial activity of the intracellular methanol extracts of (**a**) *Cladosporium cladosporioides,* (**b**) *Graminicolous helminthosporium,* and (**c**) *Bipolaris australiensis* against various bacterial pathogens. Data are represented as means ± SD of three replicates. If followed by different letters, the results are significantly different according to Duncan’s multiple range test at *p* ≤ 0.05.

Amelia et al. [33] described the antimicrobial efficacy of the ethyl acetate extract of *Penicillium* against the bacterial species of *Escherichia coli*, *Salmonella typhi*, *Staphylococcus aureus*, and *Pseudomonas aeruginosa* and the results indicated that the highest level of the zone of inhibition (13 mm) was observed against *Staphylococcus aureus*. Ding et al. [34] also reported that the antimicrobial activity was performed with the microfungal extracts of *Cladosporium sp.* Against eight different microorganisms, such as *Phoma sp.*, *Staphylococcus aureus, Sarcina lutea, Escherichia coli, Bacillus pyocyaneus, Streptomyces viridochrmogenes, Candida albicans,* and *Mucor miehei* and obtained the highest level of the zone of inhibition (25 mm) against *Streptomyces sp.* and followed by (24 mm) against *Escherichia coli* through the agar diffusion method. Similarly, we performed antimicrobial activity analysis with the ethyl acetate extract as well as the methanol mat extract of *Graminicolous helminthosporium, Bipolaris australiensis,* and *Cladosporium cladosporioides* endophytic fungi against pathogenic bacterial species, such as *Escherichia coli*, *Shigella sp*, *Streptococcus pneumoniae,* and *Streptomyces avermitilis.* The zone of inhibition pattern was different between the bacterial species as well as in the extracts used for the antimicrobial assay. For instance, the highest range of zone of inhibition (3.5 cm) was observed in the methanol mat extract of *Cladosporium cladosporioides* against *Escherichia coli* and a moderate range of zone of inhibition (1.3 cm) was observed in the ethyl acetate extract of *Cladosporium cladosporioides* against *E. coli*. The ethyl acetate and methanol mat extracts of *Bipolaris australiensis* exhibited the maximum zone of inhibition (1.9 cm) against *S. pneumoniae*. Moderate inhibition (2 cm) was noticed against *E.coli* when treated with the ethyl acetate extract and methanol mat extracts of *G. helminthosporium* (Figure 3a–c and Figure 4a–c). Similarly, Hateet [32] reported that the ethyl acetate extract of the endophytic fungus *Acremonium coenophialum* exhibited antimicrobial activity against seven Gram-positive bacteria: *S. aureus*, *Staphylococcus epidermidis*, *Streptococcus* sp., *Pneumoniae* sp., *Streptococcus pyogenes*, *Enterococcus faecalis*, *Bacillus cereus,* and *Bacillus subtilis,* which ranged between (10.0–17.0 mm) whereas the range of microbial inhibition against seven Gram-negative bacteria, namely *E.coli*, *S*. *typhi*, *P. aeruginosa*, *Klebsiella pneumoniae*, *Morganella morganii*, *Serratia* sp., and *Proteus vulgaris* was between (10.0–18.0 mm). In line with this, in this study, the ethyl acetate extract and the methanol mat extract of *G. helminthosporium, C. cladosporioides,* and *B. australiensis* revealed the zone of inhibition against *E. coli, Shigella sp, Streptocmyces avermitilis,* and *Streptococcus pneumoniae* (0.3–3.5 cm) and the results are represented in Figure 3a–c and Figure 4a–c.

### 3.5. Phytochemical Analysis of the Endophytic Microfungal Extract

Mehdi et al. [24] reported that phytochemical screening of the *Tamarindus indica* extracts revealed the presence of secondary metabolite classes that are responsible for causing the obtained antibacterial activity. Sundar et al. [23] reported investigations of the methanol mat extract and ethyl acetate extract of endophytic microfungal species of *Cladosporium* and noticed various chemical constituents, such as phenolic compounds, saponins, flavonoids, tannins, and terpenoids. In association with this, we observed various bioactive compounds, such as flavonoid, saponin, tannin, carbohydrate, phenol, terpenoid, steroids, glycoside, alkaloids, and protein in fungal extracts of both the ethyl acetate extract and methanol mat extract. The ethyl acetate and methanol extracts of *C. cladosporioides* contain a high concentration of flavonoid, phenol, and protein, whereas a higher level of tannin, carbohydrate, terpenoids, steroids, glycoside, and alkaloids were found to be observed in the methanol mat extracts. Higher levels of flavonoid, phenol, terpenoid, and steroids were observed in the ethyl acetate extracts of *Bipolaris australiensis,* while the methanol mat extracts contained the maximal level of tannin, phenol, glycoside, carbohydrate, and protein. Ethyl acetate and methanol mat extracts of *G. helminthosporium* were shown to contain increased levels of flavonoid, phenol, terpenoid, steroids, glycoside, alkaloids, and protein, whereas the methanolic extract had only a higher level of tannin and carbohydrates. Flavonoids were observed in all the extracts of endophytic fungi. On the whole, the phytochemical analysis of the extracellular and intracellular extracts of *G. helminthosporium, C. cladosporioides*, and *B. australiensis* revealed the presence of a variety of secondary metabolites, which include Flavanoids, Saponin, Tannin, Carbohydrate, Phenolic compounds and terpenoid (Table 3).

### 3.6. Antioxidant Activity of Endophytic Microfungal Extracts

#### 3.6.1. DPPH Antioxidant Scavenging Activity of Fungal Extracts

The effects of the fungal extract’s radical-scavenging abilities were investigated using DPPH, a stable free radical. Antioxidants reduce absorption by providing this radical with a proton. The reaction could be seen as a purple color turning yellow. The ethyl acetate and methanolic extracts of the mycelial mats of endophytes (*Daldinia eschscholtzii* and *Trichoderma asperellum*) isolated from *Ananus comosus* L. wereexamined for antioxidant potential in vitro using DPPH [35]. The lowest EC50 value of the free-radical scavenging activity in the ethyl acetate extract of fungal endophyte *Trichoderma asperellum* indicates a better ability of the mycelial mat extract to act as a DPPH radical scavenger. The Unknown sp. (ML5) demonstrated the least antioxidant activity, with only 33.95%, whereas the EA extract of *Alternaria* sp. (ML4) showed a high scavenging activity of 85.20%. As a benchmark, ascorbic acid demonstrated 96.91% antioxidant activity, the percentage of ascorbic acid and endophytic fungus that may scavenge DPPH radicals [36]. Similarly, an improved antioxidant activity was observed in the extracellular and intracellular extracts of *Graminicolous helminthosporium, Cladosporium cladosporioides,* and *bipolaris australiensis,* which was determined by DPPH. The highest scavenging activity was recorded in the ethyl acetate extract of *Cladosporium cladosporioides* (70.23%) and methanol extract of *Bipolaris australiensis* (95.97%) (Table 4 and Table 5). The ethyl acetate and methanol mat extracts of *B. australiensis* possess enhanced DPPH scavenging activity compared to other fungal extracts, such as *Cladosporium cladosporioides* and *Graminicolous helminthosporium*.

#### 3.6.2. H_2_O_2_ and Nitric Oxide Antioxidant Scavenging Activity of Fungal Extracts

In the analysis of H_2_O_2_ scavenging activity, the extracellular extracts of *Graminicolous helminthosporium, Bipolaris australiensis,* and *Cladosporium cladosporioides* had the maximum scavenging activity when compared to the methanol mat extract of *G. helminthosporium, B. australiensis,* and *C. cladosporioides* (Table 6 and Table 7). In particular, the extracellular extracts of *B. australiensis* and *C. cladosporioides* recorded maximum scavenging activity (96%) when compared to standard ascorbic acid. However, moderate nitric oxide scavenging activity (86%) was observed in both the ethyl acetate and methanol extracts of *Graminicolous helminthosporium, B. australiensis,* and *C. cladosporioides* (Table 8 and Table 9). In particular, the extracellular extracts of *B. australiensis* and *C. cladosporioides* have a better antioxidant scavenging percentage.

#### 3.6.3. Molecular Docking Study

**Figure 5:**
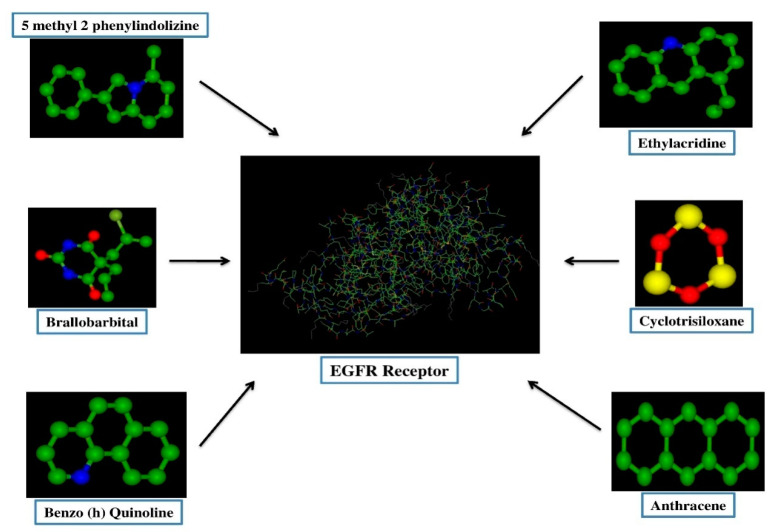
The 3D structural representation of small lung cancer receptor (EGFR) and various metabolites (ligands) identified through GC-MS from endophytic fungi, such as *Cladosporium cladosporioides, Graminicolous helminthosporium*, and *Bipolaris australiensis.*

**Figure 6:**
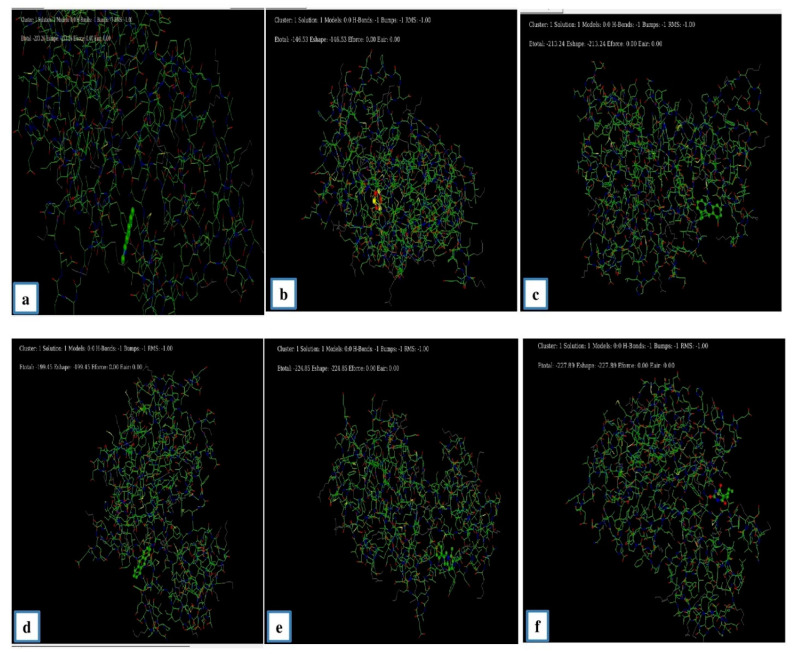
The interaction of bioactive metabolites of endophytic fungi (ligands) and the EGFR receptor. EGFR interaction with (**a**) Benzo (h) Quinoline, (**b**) Cyclotrisiloxane, (**c**) Ethylacridine, (**d**) Anthracene, and (**e**) 5-methyl 2 phenylindolizine and (**f**). Brallobarbitol.

Molecular docking was performed with the Hex 8.0 software, highly interactive software to calculate the interactions between ligands and receptors. The 3D structure of the lung cancer EGFR receptor was retrieved from the PDB database. Several studies reported that the various cancers, including NSCLC, were correlated with the EGFR mutations [8]. The 3D biostructure of the bioactive compounds (ligands), such as Benzo (h) quinolone, Ethylacridine, Cyclotrisiloxane, 5 Methyl 2 Phenylindolizine, and Brallobarbital were obtained from the analytical results of the GC-MS analysis of endophytic fungi and retrieved from the PubChem database (Figure 5). Through docking the E-minimization and E-maximization values of the receptor and ligands were calculated. Among the various bioactive compounds, the lowest E (E min) values were recorded for Brallobarbital (−227.89), and 5 methyl 2 phenylindolizine (−224.8), which suggest that these compounds might serve as potential candidates to target the EGFR receptor which is a vital signaling molecule for various types of cancers (Table 10 and Figure 6). Similarly, Meenambiga and Rajagopal [37] reported that the molecular docking with ligands of bioactive metabolites, such as Taxifolin, Saltillin and 6-methoscyflavone obtained from the endophytic fungi *Aspergillus nidulans* showed maximum binding energy against the receptor of the N-myristoyl transferase enzyme in a pathogenic fungus *Candida albicans.*

## 4. Conclusions

Three different endophytic fungi, *Cladosporium cladosporioides, Graminicolous helminthosporium*, and *Bipolaris australiensis* were isolated from various medicinal plants. The ethyl acetate (extracellular) and methanol mat (intracellular) extracts of endophytic fungi (*G. helminthosporium, B. australiensis*, and *C. cladosporioide*) were subjected to various antioxidant assays, such as DPPH, nitric oxide and H_2_O_2_ scavenging as well as antibacterial assays to determine the potential of bioactive compounds present in the extracts of endophytic fungi. Furthermore, the bioactive compounds were predicted using GC-MS, which served as ligands for in silico molecular docking of the lung cancer receptor EGFR. The extracts of endophytic fungi showed potential antioxidant activity and better antibacterial activity against several bacterial pathogens due to the presence of various bioactive compounds in the extracts identified through GC-MS. Phytochemcial composition analysis of endophytic extracts revealed the presence of various compounds, such as flavonoids, phenols, glycosides, carbohydrates, saponins, and proteins, etc. The anticancer potential of bioactive metabolites, which served as ligands was analyzed through molecular docking, which suggested that some compounds, such asBrallobarbital and 5 methyl 2 phenylindolizine had the lowest E min values that indicated these compounds may be used in anticancer drug development. However, further in vitro and molecular studies are warranted to harvest the benefits of the endophytic extracts in the drug development.

## Figures and Tables

**Table 1:** FTIR analysis of ethyl acetate (extracellular) and methanol crude (intracellular) extracts of *Gaminicolous helminthosporium*, *Bipolaris australiensis,* and *Cladosporium cladosporioides.*

Extracellular (Ethyl Acetate) Extract			Intracellular (Methanol Mat) Extract				
*Gaminicolous helminthosporium*	*Bipolaris australiensis*	*Cladosporium cladosporioides*	*Gaminicolous helminthosporium*	*Bipolaris australiensis*	*Cladosporium cladosporioides*		
Peak Range	Peak Range	Peak Range	Peak Range	Peak Range	Peak Range	Group	Compound
517.85	517.85	456.13	516.89	516.89	516.89	C-I stretching	halo compound
580.53	547.75	517.85	596.93	595.96	593.07	C-I stretching	halo compound
635.50	592.11	592.11	640.48	648.04	648.04	C-I stretching	halo compound
692.40	636.47	635.50	678.90	679.89	678.90	C-H bending	Monosubstituted
806.19	-	-	-	-	874.66	C=C bending	Alkene
947.95	814.87	-	-	1007.74	940.23	N-O stretching	Oxidized Nitrogen
1056.92	1055.95	1057.88	1081.03	1056.92	1008.70	CO-O-CO stretching	Anhydride
1171.68	1101.28	-	1148.53	1147.57	1055.95	P=O stretching	phosphine oxide
1243.04	1243.04	1243.04	1384.79	1384.79	1147.57	C-N stretching	Amine
-	1315.36	1312.26	1454.23	1315.36	1213.14	S=O stretching	Sulfonamide
1371.29	1338.51	1394.44	1338.51	1488.94	1398.30	N-O stretching	nitro compound
1644.20	1510.16	1510.16	1511.12	1589.23	1511.12	C=C stretching	α,β-unsaturated ketone
1679.88	1643.24	-	-	-	1605.63	C=O stretching	tertiary amide
1712.67	1712.67	1712.67	1717.49	-	-	C=O stretching	carboxylic acid
1767.64	1767.64	1759.92	-	1800.43	-	N=C=S stretching	Isothiocyanate
2314.42	2312.49	2360.71	2309.60	2309.60	2309.60	O=C=O stretching	carbon dioxide
2816.84	2882.42	2882.42	2884.35	2867.95	2868.92	C-H stretching	Aldehyde
2882.42	2980.78	2991.39	2981.74	2977.89	2942.21	C-H stretching	Alkane
2993.32	3290.33	-	-	3284.55	3336.62	C-H stretching	Alkane
3645.21	3588.32	3525.63	-	-	-	N-H stretching	primary amine
3746.47	3738.75	3739.72	-	3786.97	3723.32	O-H stretching	Alcohol

**Table 2:** Screening and characterization of bioactive compounds from ethyl acetate (extracellular) and methanol mat (intracellular) extracts of *Graminicolous helminthosporium, Bipolaris australiensis,* and *Cladosporium cladosporioides* in GC-MS analysis.

Compounds	Extracellular (Ethyl Acetate)	Intracellular (Methanol Mat)	Application
Benzo [h] quinoline	*Bipolaris australiensis, Cladosporium cladosporioides*	*Cladosporium cladosporioides*	Wound healing, antibacterial, antioxidant, antimalarial, antiviral, anticonvulsant, anti-HIV
2-Ethylacridine	*Graminicolous helminthosporium, Bipolarisaustraliensis, Cladosporium cladosporioides*	*Bipolaris australiensis, Cladosporium cladosporioides*	Antitumor Antioxidant
Cyclotrisiloxane	*Graminicolous helminthosporium, Bipolaris australiensis, Cladosporium cladosporioides*	*Graminicolou shelminthosporium, Bipolaris australiensis, Cladosporium cladosporioides*	Antibacterial, antiinflammatory Anticancer
Anthracene	*Bipolaris australiensis, Cladosporium cladosporioides*	-	Insecticidal, wood preservative, used as a preparation of cool-tar, fossil fuel, cosmetic, anticancer, antibacterial, anti-inflammatory
5-methyl-2-phenylindolizine	-	*Graminicolous helminthosporium, Bipolaris australiensis*	Inhibitory act against TB
Indole-2-one	*Graminicolous helminthosporium, Bipolaris australiensis*	-	Enrich metabolism Anticancer Antiviral
Tris(tert-butyldimethylsilyloxy)arsane	*Graminicolous helminthosporium, Cladosporium cladosporioides*	*Graminicoloushelminthosporium, Bipolarisaustraliensis, Cladosporium cladosporioides*	Antioxidant Antibacterial Antifungal
3-quinolinecarboxylic acid	*Graminicolous helminthosporium*	-	Antibacterial Antifungal
Benzene	*Graminicolous helminthosporium, Bipolaris australiensis*	-	Industrial manufacturing dyes, lubricants, drugs, fuel
n-Hexadeconic acid	*Bipolaris australiensis*	-	Antiinflammatory antioxidant, anticancer, Antinociceptive, cytotoxic compound
Oleic acid, Oxacyclotetradecan-2-one, Cis-Vaccenic acid	*Bipolaris australiensis*	-	Emulsifying agent, anticancer effect, reduces blood pressure, Reduces coronary heart disease (CHD), Antioxidant and antipolymerization agents, Used in asthma inhalers.
Oxacyclotetradecan-2-one	*Bipolaris australiensis*	-	Antibacterial activity
Cis-Vaccenic acid	*Bipolaris australiensis*	-	Antibacterial reduce sickle cell, reduction in the risk of coronary heart disease (CHD)
Tetrasiloxane	*Bipolaris australiensis*	*Bipolaris australiensis, Cladosporium cladosporioides*	Anticancer

**Table 3:** Phyto-chemical analysis of both extracellular (ethyl aceteate) and intracellular (methanol mat) extracts of *Graminicolous helminthosporium, Bipolaris australiensis,* and *Cladosporium cladosporioides.*

	Extracellular			Intracellular		
	*Cladosporium cladosporioides*	*Bipolaris australiensis*	*Graminicolous helminthosporium*	*Cladosporium cladosporioides*	*Bipolaris australiensis*	*Graminicolous helminthosporium*
Flavonoid	+++	+++	+++	+++	+++	+++
Saponin	++	- -	+	++	++	+
Tannin	++	- -	- -	+++	+++	+++
Carbohydrate	- -	- -	- -	++	+++	++
Phenol	+++	+++	+++	+	+	+++
Terpenoid	- -	+++	+++	+	++	++

**Table 4:** DPPH antioxidant scavenging activity of the ethyl acetate (extracellular) extracts of *Cladosporium cladosporioides, Graminicoloushelminthosporium*, and *Bipolarisaustraliensis.* Data are represented as means ± SD of three replicates. Numbers within a column followed by the same alphabet letters are not significantly different according to Duncan’s multiple range test at *p* ≤ 0.05.

Concentration of Samples (µL)	*C. cladosporioides*		*G. helminthosporium*		*B. australiensis*	
	Absorption Value (OD)	% of Scavenging	Absorption Value (OD)	% of Scavenging	Absorption Value (OD)	% of Scavenging
10	0.2	68.99 ^c^	0.209	67.6 ^c^	0.195	69.76 ^c^
30	0.195	69.76 ^b^	0.192	70.23 ^b^	0.192	70.23 ^b^
50	0.192	70.23 ^a^	0.187	71 ^a^	0.187	71 ^a^

**Table 5:** DPPH antioxidant scavenging activity of the intracellular extract of *Cladosporium cladosporioides, Graminicolous helminthosporium*, and *Bipolaris australiensis*. Data are represented as means ± SD of three replicates. Numbers within a column followed by the same letters are not significantly different according to Duncan’s multiple range test at *p* ≤ 0.05.

Concentration of Samples (µL)	*C. cladosporioides*		*G. helminthosporium*		*B. australiensis*	
	Absorption Value (OD)	% of Scavenging	Absorption Value (OD)	% of Scavenging	Absorption Value (OD)	% of Scavenging
10	0.187	70.01 ^c^	0.196	69.61 ^c^	0.132	79.53 ^c^
30	0.167	74.11 ^b^	0.189	70.7 ^b^	0.105	83.72 ^b^
50	0.147	77.21 ^a^	0.182	71.78 ^a^	0.026	95.97 ^a^

**Table 6:** H_2_O_2_ antioxidant scavenging activity of the ethyl acetate (extracellular) extract of *Cladosporium cladosporioides, Graminicolous helminthosporium*, and *Bipolaris australiensis.* Data are represented as means ± SD of three replicates. Numbers within a column followed by the same letters are not significantly different at *p* ≤ 0.05.

Concentration of Samples (µL)	*C. cladosporioides*		*G. helminthosporium*		*B. australiensis*	
	Absorption Value (OD)	% of Scavenging	Absorption Value (OD)	% of Scavenging	Absorption Value (OD)	% of Scavenging
10	0.123	93.35 ^b^	0.17	90.82 ^c^	0.167	90.98 ^c^
30	0.092	95.01 ^a^	0.118	93.63 ^b^	0.091	95.03 ^b^
50	0.087	95.31 ^a^	0.06	96.76 ^a^	0.058	96.87 ^a^

**Table 7:** H_2_O_2_antioxidant scavenging activity of the intracellular methanolic extract of *Cladosporium cladosporioides, Graminicolous helminthosporium*, and *Bipolaris australiensis.* Data are represented as means ± SD of three replicates. Numbers within a column followed by the same letters are not significantly different according to Duncan’s multiple range test at *p* ≤ 0.05.

Concentration of Samples (µL)	*C. cladosporioides*		*G. helminthosporium*		*B. australiensis*	
	Absorption Value (OD)	% of Scavenging	Absorption Value (OD)	% of Scavenging	Absorption Value (OD)	% of Scavenging
10	0.266	85.63 ^c^	0.255	86.23 ^b^	0.273	85.25 ^c^
30	0.213	88.49 ^b^	0.203	29.03 ^c^	0.194	89.52 ^b^
50	0.107	94.23 ^a^	0.161	91.3 ^a^	0.15	91.9 ^a^

**Table 8:** Nitric oxide antioxidant scavenging activity of the ethyl acetate (extracellular) extract of *Cladosporium cladosporioides, Graminicolous helminthosporium*, and *Bipolaris australiensis*. Data are represented as means ± SD of three replicates. Numbers within a column followed by the same letters are not significantly different according to Duncan’s multiple range test at *p* ≤ 0.05.

Concentration of Samples (µL)	*C. cladosporioides*		*G. helminthosporium*		*B. australiensis*	
	Absorption Value (OD)	% of Scavenging	Absorption Value (OD)	% of Scavenging	Absorption Value (OD)	% of Scavenging
10	0.065	86.82 ^c^	0.071	85.6 ^c^	0.068	86.21 ^b^
30	0.061	87.63 ^b^	0.065	86.82 ^b^	0.064	87.01 ^a^
50	0.059	88.03 ^a^	0.063	87.22 ^a^	0.061	87.63 ^a^

**Table 9:** Nitric oxide antioxidant scavenging activity of the intracellular methanolic extract of *Cladosporium cladosporioides, Graminicolous helminthosporium*, and *Bipolaris australiensis*. Data are represented as means ± SD of three replicates. Numbers within a column followed by the same letters are not significantly different according to Duncan’s multiple range test at *p* ≤ 0.05.

Concentration of Samples (µL)	C. cladosporioides		G. helminthosporium		B. australiensis	
	Absorption Value (OD)	% of Scavenging	Absorption Value (OD)	% of Scavenging	Absorption Value (OD)	% of Scavenging
10	0.273	44.62 ^c^	0.193	60.85 ^c^	0.32	35.1 ^c^
30	0.239	51.52 ^b^	0.073	85.19 ^a^	0.141	71.4 ^b^
50	0.065	86.82 ^a^	0.08	83.77 ^b^	0.092	81.34 ^a^

**Table 10:** E-value scoring pattern of EGFR Receptor and endophytic fungal metabolites (Ligands) obtained through molecular docking study.

Ligand	E min
Benzo [h] quinolone	−213.20
Cyclotrisiloxane	−146.53
Ethylacridine	−213.24
Anthracene	−199.45
5 methyl 2 phenylindolizine	−224.85
Brallobarbital	−227.89

## Data Availability

Not applicable.

## Supplementary Materials

The following are available online at https://www.mdpi.com/article/10.3390/antibiotics11111533/s1, Figure S1: GC-MS spectral analysis of the extracellular ethyl acetate extract of *Bipolaris australiensis*. The corresponding peaks for the bioactive compounds showed in arrow marks; Figure S2: GC-MS spectral analysis of the intracellular methanolic extract of *Bipolaris australiensis*. The corresponding peaks for the bioactive compounds showed in arrow marks; Figure S3: GC-MS analysis of the extracellular ethyl acetate extract of *Cladosporium cladosporioides*. The corresponding peaks for the bioactive compounds showed in arrow marks; Figure S4: GC-MS analysis of the methanolic intracellular mat extract of *Cladosporium cladosporioides*. The corresponding peaks for the bioactive compounds showed in arrow marks; Figure S5: GC-MS analysis of the extracellular ethyl acetate extract of Graminicolous helminthosporium. The corresponding peaks for the bioactive compounds showed in arrow marks; Figure S6: GC-MS analysis of the intracellular methanolic mat extract of Graminicolous helminthosporium. The corresponding peaks for the bioactive compounds showed in arrow marks.
