# Supplementary material for: Bioactive Components and Health Potential of Endophytic Micro-Fungal Diversity in Medicinal Plants
Source: Antibiotics (Basel). 2022 Nov 2;11(11):1533. doi: 10.3390/antibiotics11111533 (PMC9686567; doi:10.3390/antibiotics11111533)
Supplement: Supplementary file 1 [file antibiotics-11-01533-s001.zip › antibiotics-1958564-supplementary.pdf]

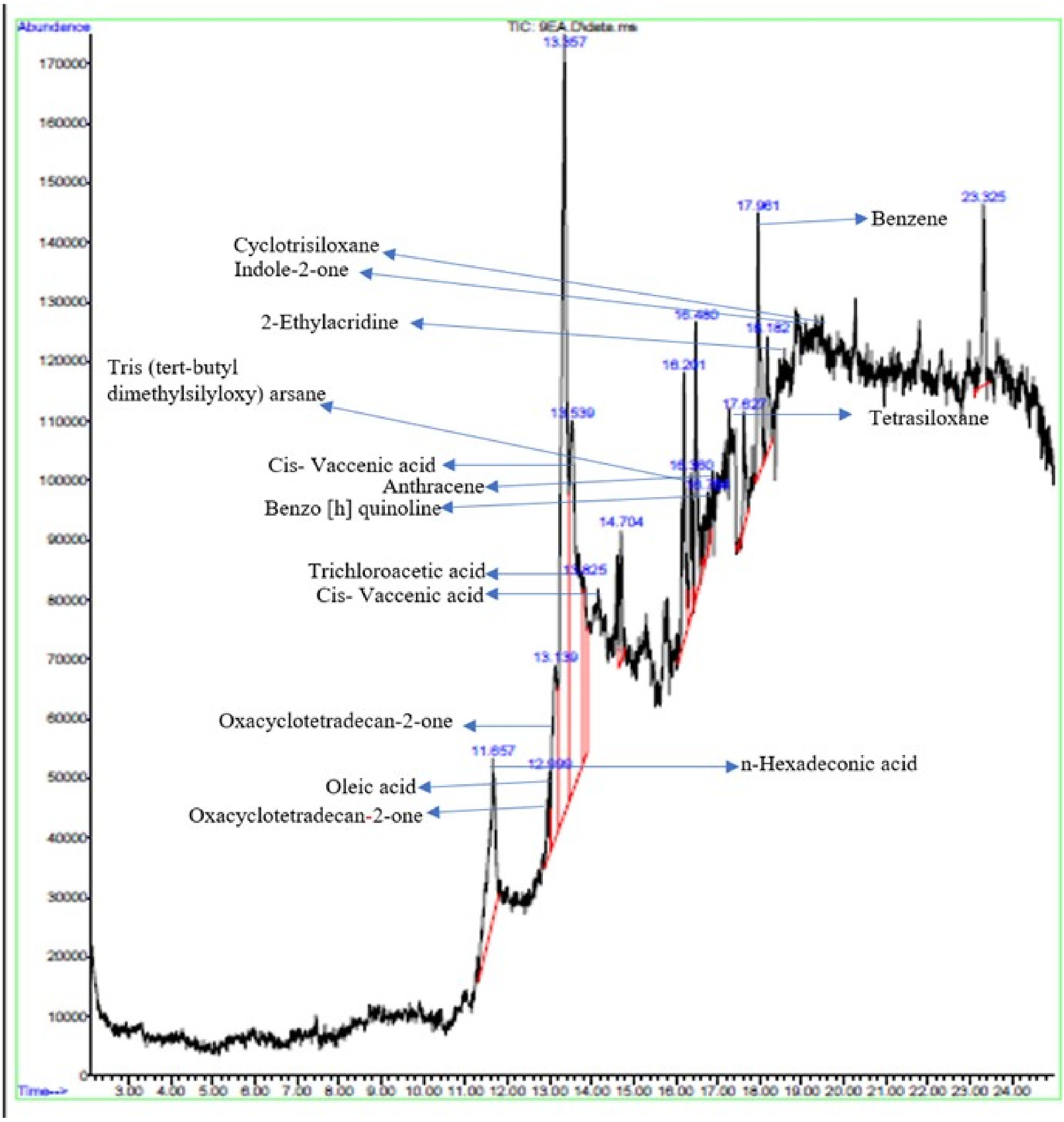


Figure S1. GC-MS spectral analysis of the extracellular ethyl acetate extract of *Bipolaris australiensis*. The corresponding peaks for the bioactive compounds showed in arrow marks.

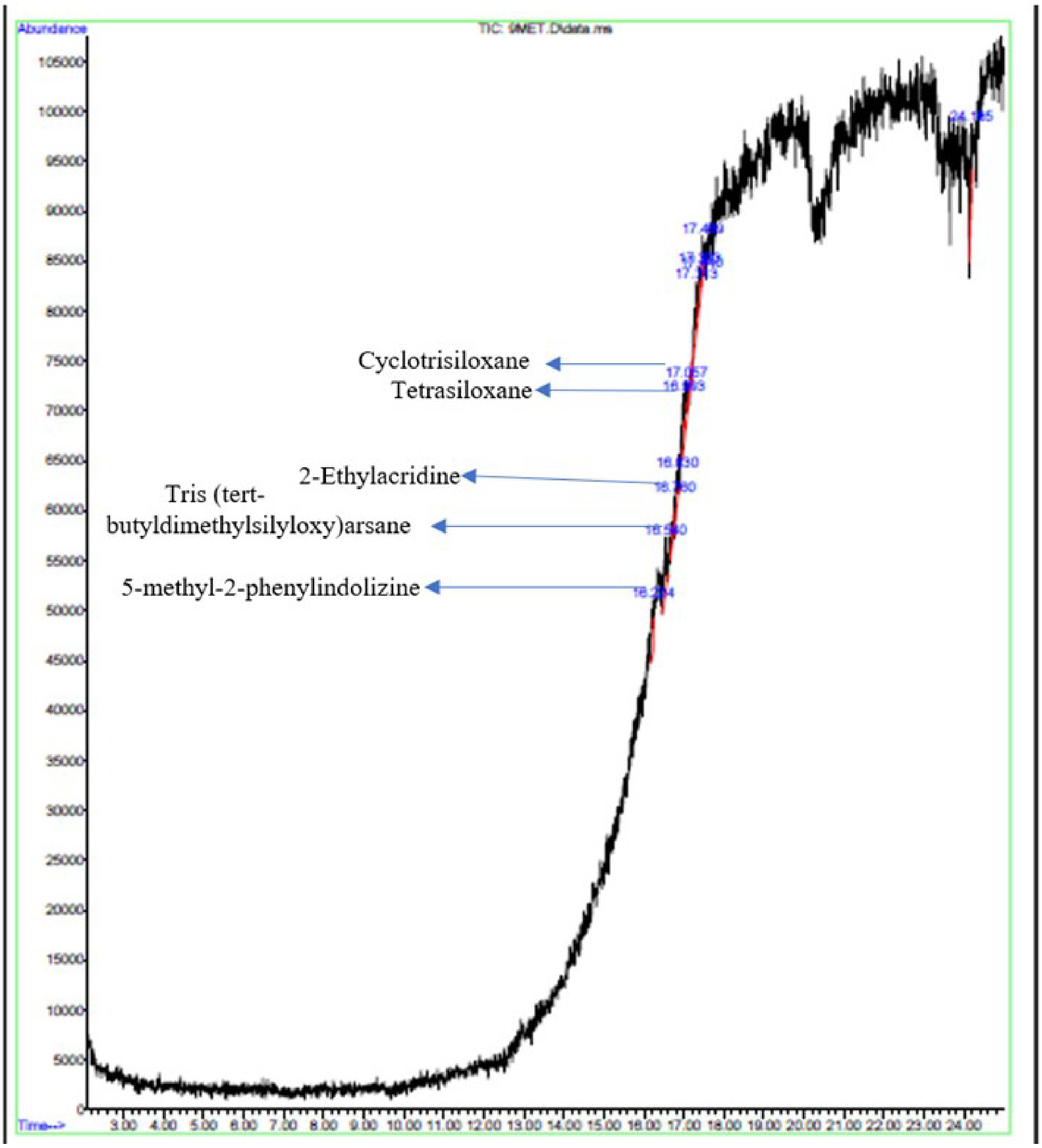


Figure S2.GC-MS spectral analysis of the intracellular methanolic extract of *Bipolaris australiensis*. The corresponding peaks for the bioactive compounds showed in arrow marks.

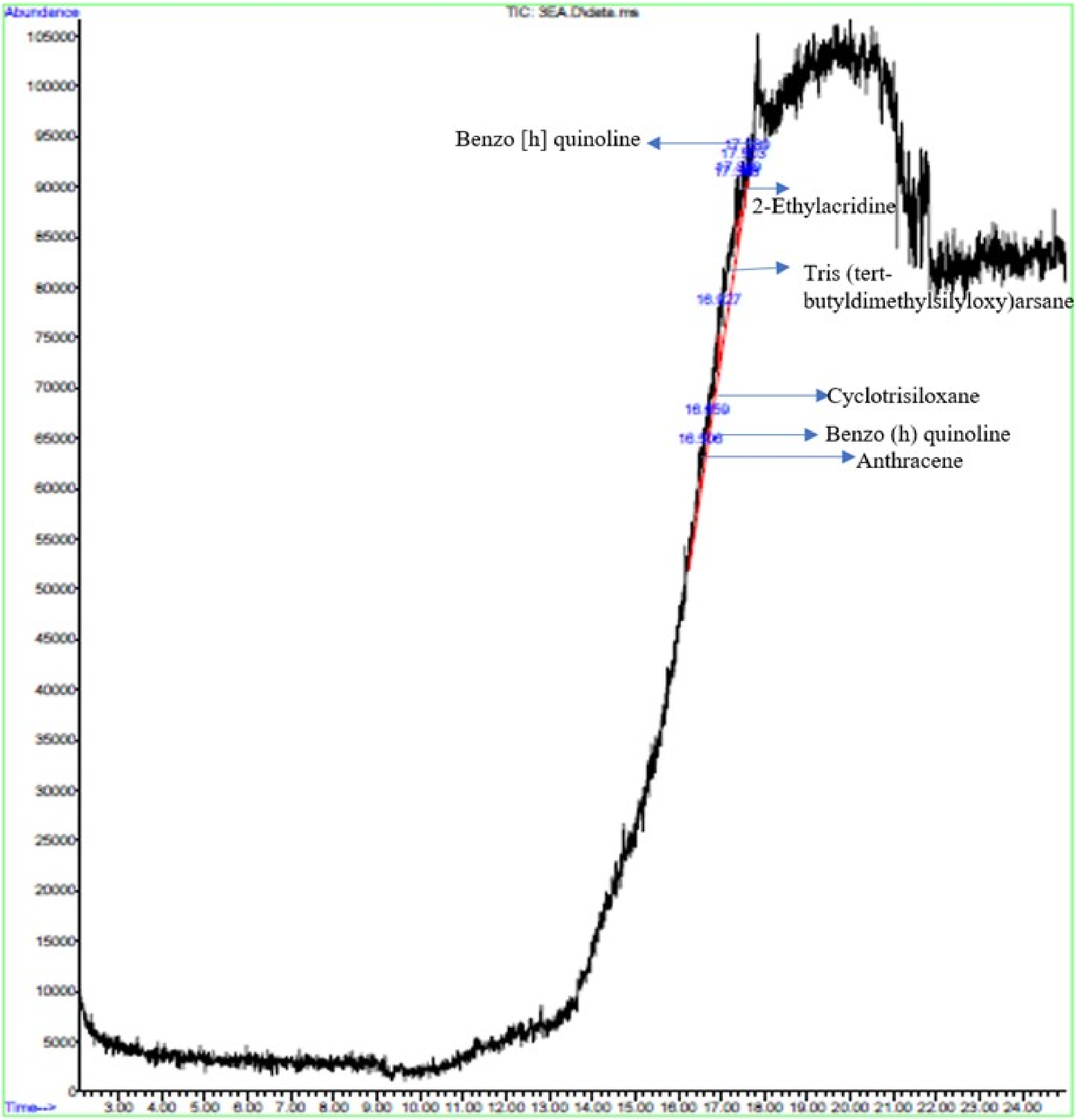


Figure S3. GC-MS analysis of the extracellular ethyl acetate extract of *Cladosporium cladosporioides*. The corresponding peaks for the bioactive compounds showed in arrow marks.

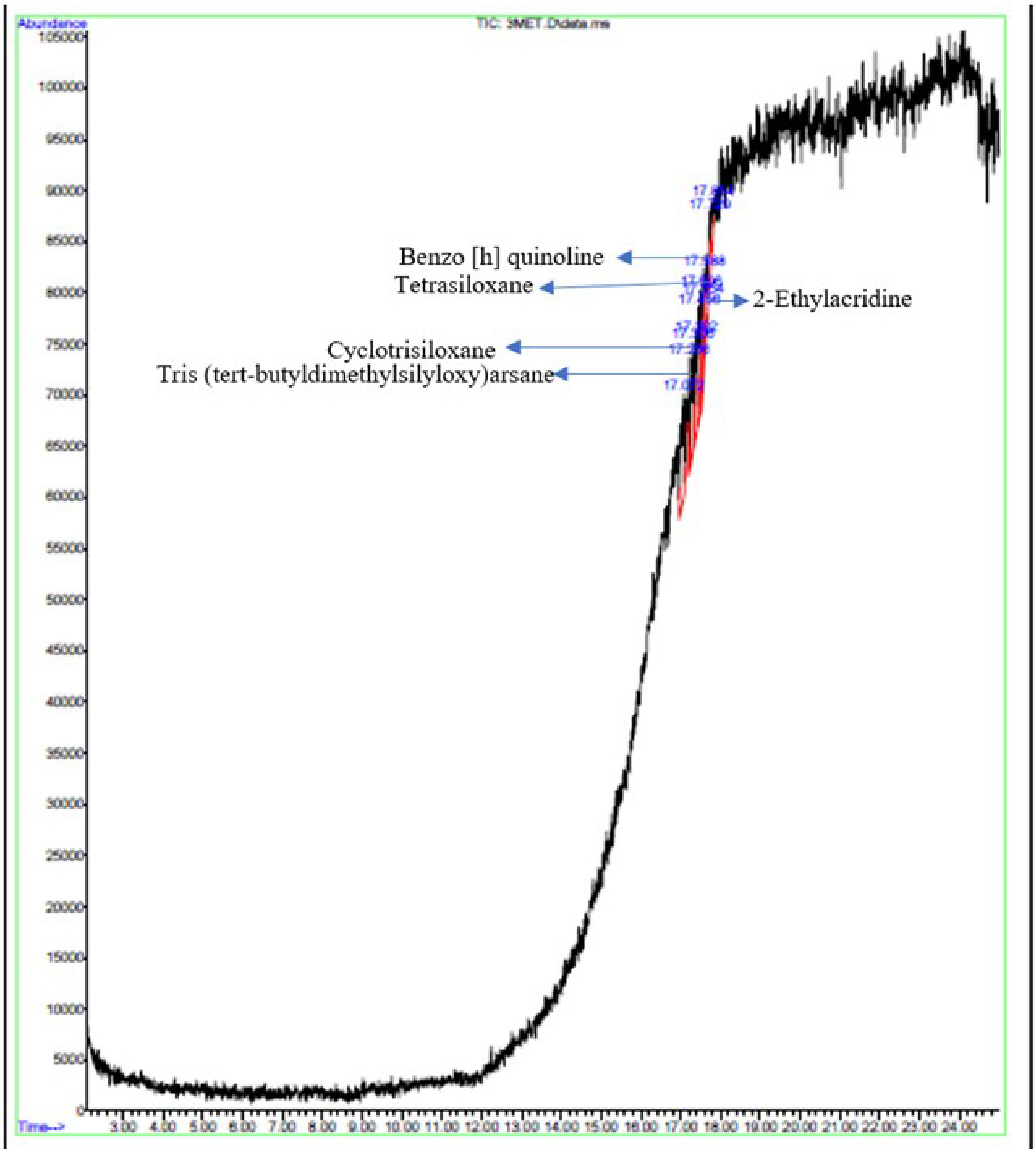


Figure S4. GC-MS analysis of the methanolic intracellular mat extract of *Cladosporium cladosporioides*. The corresponding peaks for the bioactive compounds showed in arrow marks.

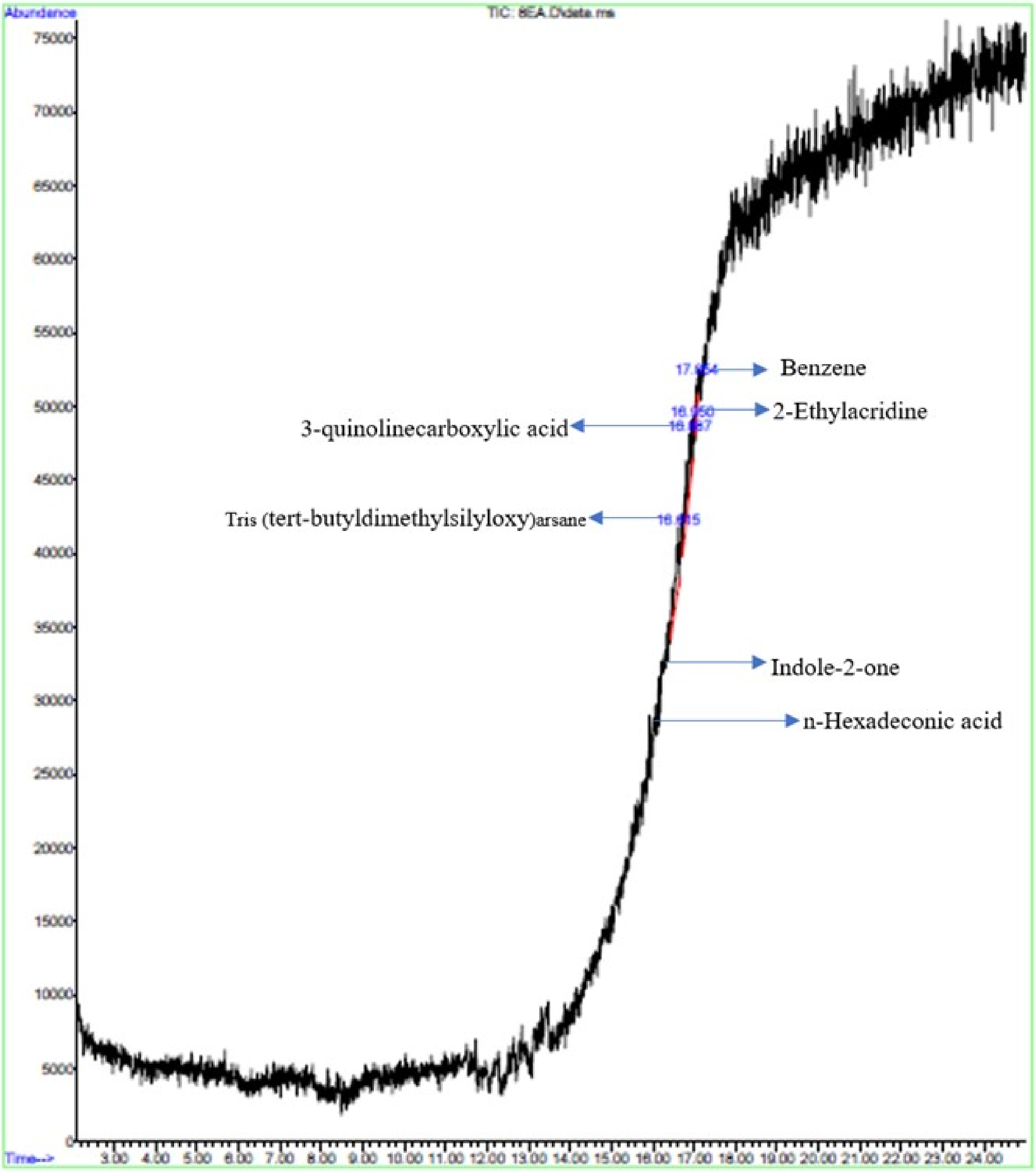


Figure S5. GC-MS analysis of the extracellular ethyl acetate extract of *Graminicolous helminthosporium*. The corresponding peaks for the bioactive compounds showed in arrow marks.

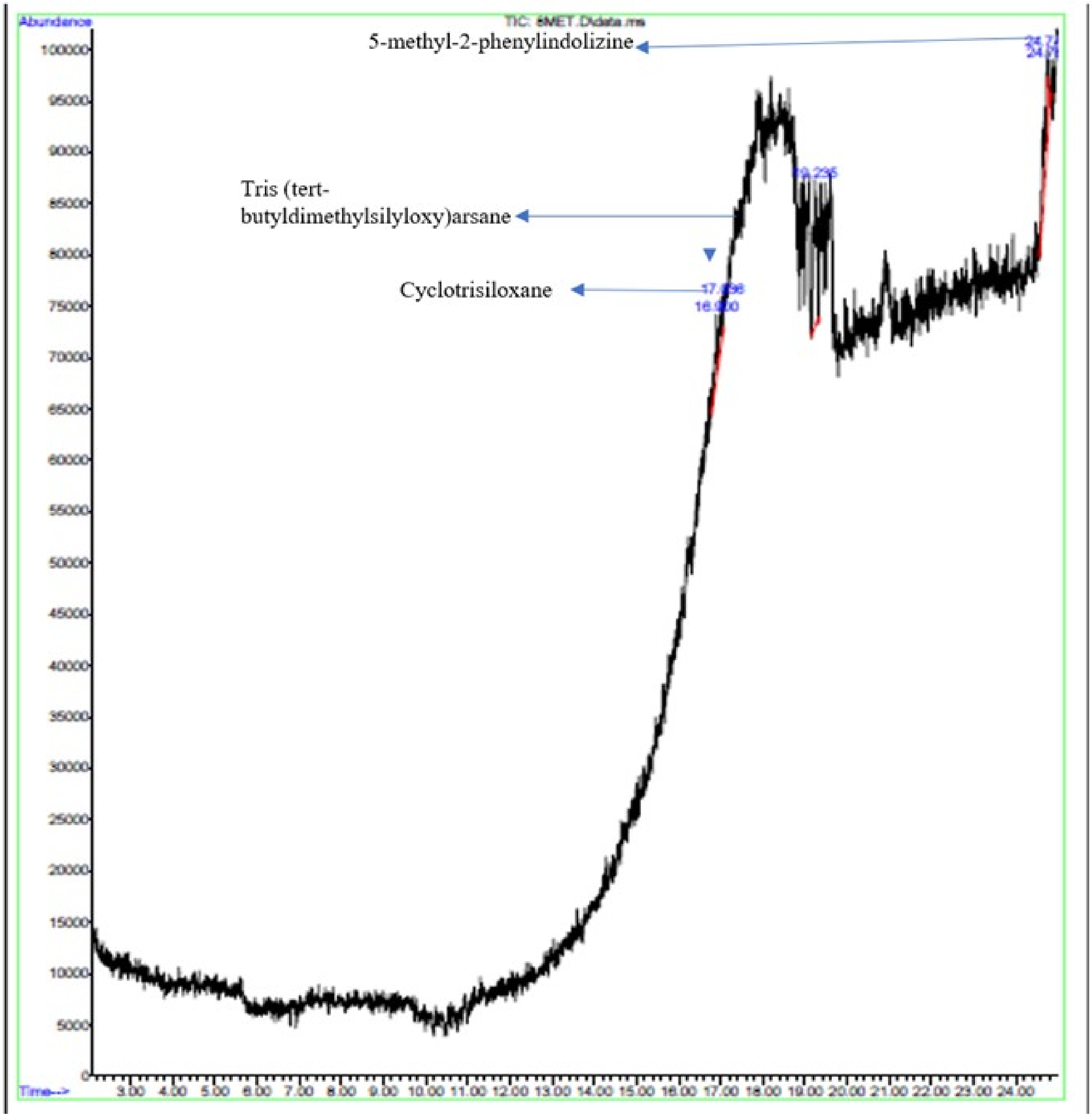


Figure S6. GC-MS analysis of the intracellular methanolic mat extract of *Graminicolous helminthosporium.* The corresponding peaks for the bioactive compounds showed in arrow marks.
